# Augmenting Flexnerism Via Twitterism: Need for Integrating Social Media Application in Blueprinting Pedagogical Strategies for Undergraduate Medical Education

**DOI:** 10.2196/12403

**Published:** 2019-03-25

**Authors:** Yajnavalka Banerjee, Richa Tambi, Mandana Gholami, Alawi Alsheikh-Ali, Riad Bayoumi, Peter Lansberg

**Affiliations:** 1 Department of Basic Medical Sciences College of Medicine Mohammed Bin Rashid University of Medicine and Health Sciences Dubai United Arab Emirates; 2 Centre for Medical Education School of Medicine University of Dundee Dundee United Kingdom; 3 Academic Medical Center, Dubai Healthcare City Department of Basic Medical Sciences Mohammed Bin Rashid University of Medicine and Health Sciences Dubai United Arab Emirates; 4 Laboratory for Computational Molecular Design RIKEN Center for Biosystems Dynamics Research Osaka Japan; 5 Academic Medical Center, Dubai Healthcare City Bachelor of Medicine & Bachelor of Surgery Program Mohammed Bin Rashid University of Medicine and Health Sciences Dubai United Arab Emirates; 6 Molecular Genetics Section Department of Pediatrics University Medical Center Groningen Groningen Netherlands

**Keywords:** social media, medical education, twitter messaging, Web 2.0, curriculum

## Abstract

**Background:**

Flexnerism, or “competency-based medical education,” advocates that formal analytic reasoning, the kind of rational thinking fundamental to the basic sciences, especially the natural sciences, should be the foundation of physicians’ intellectual training. The complexity of 21st century health care requires rethinking of current (medical) educational paradigms. In this “Millennial Era,” promulgation of the tenets of Flexnerism in undergraduate medical education requires a design and blueprint of innovative pedagogical strategies, as the targeted learners are millennials (designated as generation-Y medical students).

**Objective:**

The aim of this proof-of-concept study was to identify the specific social media app platforms that are selectively preferred by generation-Y medical students in undergraduate medical education. In addition, we aimed to explore if these preferred social media apps can be used to design an effective pedagogical strategy in order to disseminate course learning objectives in the preclinical phase of a spiral curriculum.

**Methods:**

A cross-sectional survey was conducted by distributing a 17-item questionnaire among the first- and second-year medical students in the preclinical phase at the Mohammed Bin Rashid University of Medicine and Health Science.

**Results:**

The study identified YouTube and WhatsApp as the social media app platforms preferred by generation-Y medical students in undergraduate medical education. This study also identified the differences between female and male generation-Y medical students in terms of the use of social media apps in medical education, which we believe will assist instructors in designing pedagogical strategies to integrate social media apps. In addition, we determined the perceptions of generation-Y medical students on the implementation of social media apps in medical education. The pedagogical strategy designed using social media apps and implemented in the Biochemistry course was well accepted by generation-Y medical students and can be translated to any course in the preclinical phase of the medical curriculum. Moreover, the identified limitations of this study provide an understanding of the gaps in research in the integration of social media apps in a medical curriculum catering to generation-Y medical students.

**Conclusions:**

21st century medical education requires effective use of social media app platforms to augment competency-based medical education: Augmentation of Flexnerism in the current scenario is possible only by the adaptation of Twitterism.

## Introduction

### Background

Abraham Flexner, a research scholar at the Carnegie Foundation for the Advancement of Teaching, shouldered the responsibility of pursuing an appraisal of medical education in North America, calling on all 155 medical schools in the United States and Canada. Flexner published his findings in 1910 in the form of a formal report. This report, generally referred to as the “Flexner Report,” initiated a so-called “renaissance” in global medical education through the effectuation of “competency-based medical education,” also known as “Flexnerism” [1]. Flexnerism advocates that formal analytic reasoning, the kind of rational thinking fundamental to the basic sciences, especially the natural sciences, should be the foundation of physicians’ intellectual training [2,3].

Before the advent of Flexnerism, little to no attention was devoted to the idea of whether factual knowledge was required for the expansion of higher cognitive or metacognitive abilities or if the translation of this knowledge could be applied to patient care [4,5].

However, the complexity of 21st-century health care requires rethinking of current (medical) educational paradigms, since a sizeable majority of undergraduate and graduate medical trainees today hail from the “Millennial Generation,” defined as persons born between the years 1982 and 2000 [6,7]. Millennials, also referred to as “digital natives” [8], the “instant messaging generation” [9], the “trophy kids” [10], and generation Y [11] are technologically informed, assertive, and, oddly enough, motivated by self-interest, yet intensely altruistic in sharing their personal information [12]. They are familiar with and able to integrate rapidly evolving technologies into every aspect of their lives. Therefore, generation Y medical students have a one-off outlook on education and different inclinations and expectancies than their forerunners [13]. Consequently, new pedagogical strategies need to be strategized, and the existing ones need to be fine-tuned or perfected, such that they can appeal to generation-Y medical students as well as concomitantly address the demands of the 21st century medical education paradigms. In other words, pedagogical strategies such as didactic teaching techniques need to be augmented by generation-Y medical student–centric pedagogical strategies and implementation milieus [14]. Thus, dissemination strategies of Flexnerism need to evolve and keep pace with the Millennial age Zeitgeist.”

Social media apps refer to “websites and applications that enable users to create and share content, to interact with other users or to find people with similar interests.” The term covers multiple platforms encompassing blogs/micro blogs (Twitter), Wikis, YouTube, and social network sites such as Facebook [15]. Integration of social media apps in pedagogical strategies for generation-Y medical students will not only address their learning needs but also appeal to the generation-Y medical students ’ preferred learning style or strategy. The rationale for this is supported by the learning theory of connectivism.

Connectivism considers learning a multifaceted process catalyzed by technology and socialization [16]. The foundations of connectivism are fueled by chaos, connectivity, complexity, and self-organization theories. According to Downes, connectivism also finds its roots in connectionism, associationism, and graph theory [17]. The principles of connectivism, per Siemens [16,18], are as follows:

- Learning and knowledge rest in the diversity of outlook.
- Learning is a process of connecting specialized nodes or information sources.
- Learning may reside in nonhuman appliances.
- Capacity to know is more critical than what is currently known.
- Nurturing and maintaining connections are needed to facilitate continual learning.
- Ability to see connections between fields, ideas, and concepts is a core skill.
- Currency (accurate and up-to-date knowledge) is the intent of all connectivist learning activities.
- Decision making is itself a learning process. Choosing what to learn and the meaning of incoming information is seen through the lens of a shifting reality. Although there is a right answer now, it may be wrong tomorrow due to alterations in the information climate affecting the decision.

Therefore, instructors, in a connectivist learning milieu, guide students to information (that can preferably be accessed with ease) and address queries as required, to encourage students’ learning and sharing on their own accord through the creation of a learning community. Students are also spurred to seek out information on the Web, critique the information, and share their findings and opinions within the learning community that they have created.

### Connectivism Through a Social Media App–Integrated Pedagogical Strategy

A working example of a pedagogical strategy founded on connectivist learning principles through social media apps targeting generation-Y medical students is presented below. In such a strategy, the instructor would perform the following tasks:

- Present the intended learning outcomes for a specific topic
- Conduct a short-didactic session to disseminate the baseline knowledge with regard to the intended learning outcomes
- Ask students to network and create a Learning Community using social media apps of their choice, where the instructor is also added
- Elaborate on the disseminated knowledge by directing students to social media app resources, where such information is available
- Encourage students to share and critique the information in the Learning Community using the social media app platform identified earlier
- Follow the discussion as a participant and address students’ queries

However, with the plethora of social media app platforms available, it is essential to identify apps that should be employed in the design of generation-Y medical student–targeted pedagogical strategies. However, instead of instructors identifying a social media app for such a purpose, platforms preferred by the generation-Y medical students should be selected, *so that the creation of the Learning Community is facilitated and embraced*.

### Study Landscape

We performed a proof-of-concept study at the College of Medicine and Health Sciences at the Mohammed Bin Rashid University (MBRU) to identify social media app platforms that should be employed/integrated in the design of generation-Y medical student–targeted pedagogical strategies as well as the perception of generation-Y medical students of the benefit of social media apps on different facets of undergraduate medical education. Our study involved students in the preclinical years, as this phase of undergraduate medical education in an integrated curriculum (refer below) builds up the corpus of knowledge that is translated to diagnosis and patient care in the clinical years. The obtained results primarily indicate that specific social media app platforms are preferred by generation-Y medical students in the undergraduate medical curriculum, integration of which in the design of pedagogical strategies will lead to favorable outcomes. We also present an example from the course of Biochemistry for undergraduate medical students at MBRU, where integration of social media apps in pedagogical strategies resulted in the positive feedback from generation-Y medical students.

## Methods

### Study Milieu

MBRU is a new medical school, where the curriculum is founded on a competency-based educational model [19], in line with the tenets of Epstein and Hundert [20,21], and spans over 6 years. Therefore, the MBRU curriculum provides a milieu for a highly adaptive learning process rather than the traditional “one-size-fits-all curriculum” [22]. Furthermore, the MBRU curriculum aims to foster an erudition environment, where peer-assisted learning [23] and learning supported by social learning theories are facilitated.

**Figure 1:**
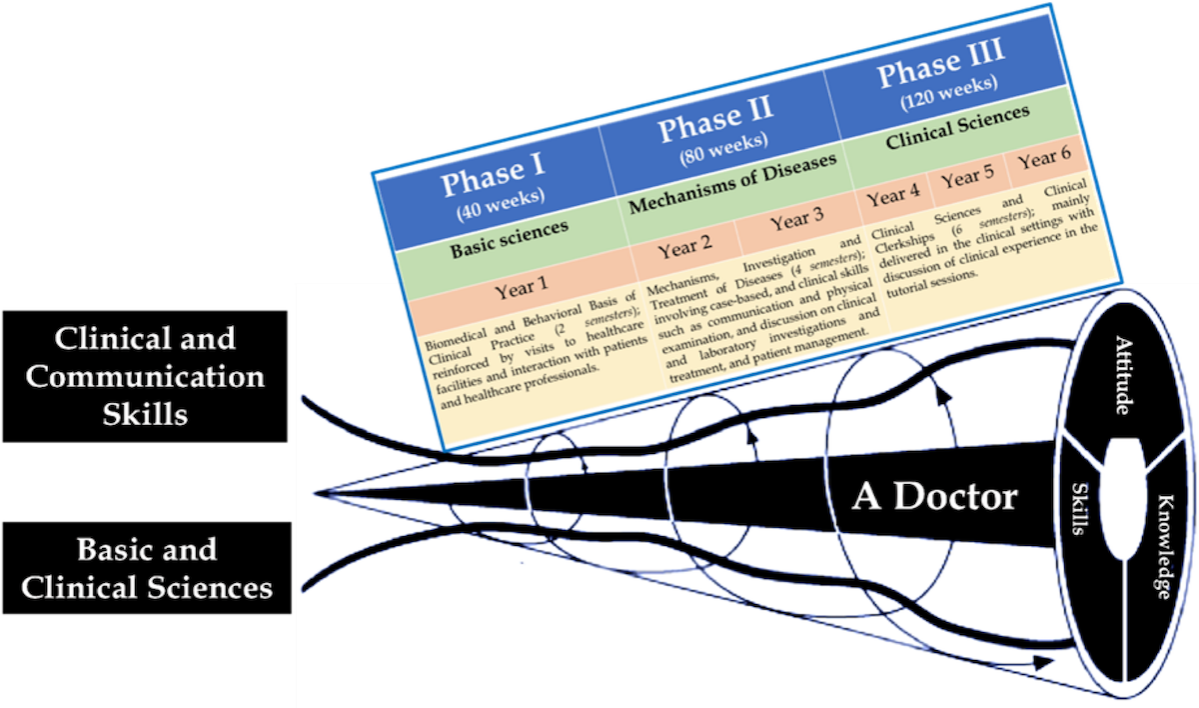
The undergraduate medical curriculum at Mohammed Bin Rashid University of Medicine and Health Sciences. The curriculum is divided into three phases and spans over 6 years. Each phase of the undergraduate medical curriculum includes integrated courses and builds on the preceding one, such that the curriculum is a “spiral,” and the students repeat the study of a subject, each time at a higher level of difficulty and in greater depth.

The MBRU curriculum is divided into 3 phases (Figure 1). Each phase of MBRU’s MBBS curriculum includes integrated courses and builds on the preceding one, such that the curriculum is “spiral” and the students repeat concepts pertaining to a subject, where with each successive encounter, concepts build on the previous one. (Figure 1).

Hence, integration of social media apps in pedagogical approaches in such a curriculum is essential to facilitate and augment sharing of information, where learning will be guided by social constructivist principles of Dewey and Vygotsky, a variation of cognitive constructivism that emphasizes the need for collaboration in the learning process [24].

As our institution is in its third year of operation, where the preclinical phase is nearing completion, we believe it would be pivotal to identify social media app platforms preferred by generation-Y medical students as well as their perception on the use of generation-Y medical students in medical education, as courses can be updated to incorporate innovative pedagogical strategies where social media apps are used, such that future students benefit from this update.

Furthermore, the results of our study will benefit students in their clinical years, as the obtained results will allow instructors in the clinical phase to design innovative social media app–integrated pedagogical strategies to deliver the intended learning outcomes.

### Approach

#### Data Collection

A blueprint of an online questionnaire was developed with cues from a questionnaire that has been used for a similar study, with modifications following gap analysis (see Table 1 for details and Multimedia Appendix 1 for the questionnaire) [25-27].

The study was approved by the institutional review board. Google forms were used to design the survey and collect data. The link to this form was distributed among the first- and second-year medical students in the preclinical phase of the curriculum at MBRU, through WhatsApp, Gmail, and Facebook Messenger, after obtaining prior consent from the participants between March 2018 and May 2018.

#### Questionnaire Design

The questionnaire was followed by an initial cover letter, which explained the purpose of the study to generation-Y medical students. It consisted of 17 questions followed by an optional comment section (overall response of the students to the comments section is summarized in Multimedia Appendix 2).

**Table 1:** Results of the gap analysis conducted through a literature review of seminal articles for designing the questionnaire.

Reference (year)	Title	Publication year	Abstract summary
Durga et al [28]	Social media: Portrait of an emerging tool in medical education	2016	The article examines social media use in medical education in commentaries and descriptive accounts versus evaluative studies to compare the relative prevalence of the two themes, challenges, and opportunities of social media in this context. The outcome reported a higher prevalence of challenges in commentaries and descriptive accounts.
Pander et al [29]	The use of Facebook in Medical Education	2014	This systematic review explicitly explores Facebook and its incorporation in Medical Education. Results indicate that most studies have looked into Facebook and digital professionalism. It has been well accepted among students for use for various learning interventions. Nonetheless, the study reports the absence of evidence to assess the effectiveness of Facebook as a learning method in advanced stages.
Hollinderbäumer et al [30]	Education 2.0—How has social media and Web 2.0 been integrated into medical education? A systematical literature review	2013	In this systematic literature review, the authors assessed how the combined use of social media and Web 2.0 has been implemented in medical education. It illustrated ways to enhance student reflection and allowed students to advance their knowledge.
Cartledge et al [31]	The use of social-networking sites in medical education	2013	This article focuses on the success rate of social media network sites in delivering educational content and determines if health care professionals and students utilize these sites for educational purposes. The article concluded that no professionalism issues arose with implementation of social networking sites and the study was received positively. However, it stated that there is not enough evidence to support the relative effectiveness of social networking sites over traditional methods.
Cheston et al [32]	Social media use in Medical Education: A systematic review	2013	This article aims to address the following questions: (1) How have interventions using social media tools affected outcomes of satisfaction, knowledge, attitudes, and skills for physicians and physicians-in-training? (2) What challenges and opportunities specific to social media have educators encountered in implementing these interventions? The results showed that implementation of social media contributed to enhanced outcomes of the elements mentioned in Question 1. The most common opportunity regarding implementation of social media was stimulating active learner engagement, and the most commonly faced challenge was technical issues.

The questionnaire focused on the different ways in which social media apps affect the education of medical students. These 17 questions can be classified into 4 groups:

1. *Group I questions* consisted of items that were related to students’ consent and their personal information (year and gender).
2. *Group II questions* consisted of 5 questions related to the frequency of use of social media apps and the preferred social media app platform.
3. *Group III questions* comprised 7 questions regarding generation-Y medical students’ perception about the effect of social media apps on their learning, communication, and association with regard to medical education.
4. *Group IV questions* included 2 questions in the questionnaire, which provided insight into generation-Y medical students ’ awareness of the ethical issues associated with the use of social media apps.

The questionnaire was vetted by all the authors. Before circulating the questionnaire via the Google Forms Link, a mock Google Forms link was circulated to year 1 and year 2 generation-Y medical students at MBRU in order to ensure that their smart devices were compatible with the survey platform.

Respondents had to submit their responses before a specific deadline, which was set at 10 weeks after circulation of the questionnaire.

#### Statistical Analysis

Responses were drawn directly from Google Form into an Excel file. Descriptive statistical analysis was performed using Microsoft Excel (Version 14, Microsoft Corporation, 2010).

## Results

### Participants

A total of 75 MBRU students participated, of which 8 were excluded: 6 did not complete the questionnaire and 2 did not use social media apps in their education. Data collected from the remaining 67 students were further analyzed.

The study population included 48 female students and 19 male students, of which 61% (41/67) of the respondents were in the second year of medical school. In both year 1 and year 2, majority of the students (>69%) were female. This trend of having more female students in both year 1 and year 2 is not unusual. In fact, female students now outnumber male students in most medical schools in a ratio of about 3:2 [33].

However, despite this upturn in the number of female students, there are still insufficient women in some areas, especially clinical academia. The United Kingdom Medical Schools Council report of 2007 indicates that only 11% of the professorial staff in UK medical schools and 36% of clinical lecturers are women. The proportion of women decreases with increasing academic grade. An analogous situation exists in the United States, where only 15% of full professors and 11% of department chairs are women [34], despite of the fact that several recent studies of leadership indicate that women are good at inspiring others and virtuous team leaders [35]. In addition, women are not represented equally across the profession, especially in specialties necessitating more critical and on-call responsibilities and more technical skills [36]. Can this disparity be attributed to the way women learn, because positions associated with increased academic and administrative responsibilities require a more tailored pedagogical style specifically for the augmentation of metacognitive skills?

A study by Wehrwein et al showed that a majority of male students preferred multimodal instruction, specifically, 4 modes conveyed as “VARK”: visual (V; learning from graphs, charts, and flow diagrams), auditory (A; learning from speech), read-write (R; learning from reading and writing), and kinesthetic (K; learning from touch, hearing, smell, taste, and sight). In contrast, a majority of female students were tuned to single-mode instruction with a preference toward K [37].

Traditional pedagogical approaches such as the Sage on the Stage method involving didactic teaching cannot be tailored for preferential teaching/learning. However, a social media app–incorporated pedagogical approach can be tailored and moderated by both the instructor and learner *.* As the majority of our respondents are female, this study brings highlights the social media app preference of female generation-Y medical students, which, if suitably adapted in the design of pedagogical approaches, will cater to the learning needs/preferences of women in medical education, assuaging the gender disparity in the different domains of medicine in the long run.

### Frequency of Social Media App Use by Generation-Y Medical Students for Medical Education

**Figure 2:**
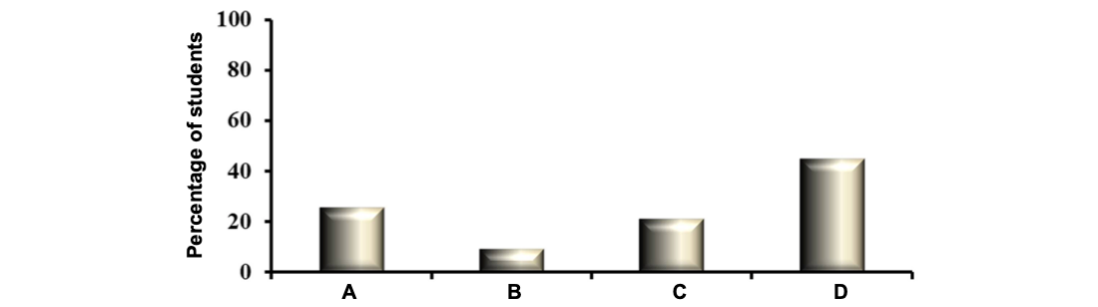
The frequency of use of social media application by respondents in the study. The number of students in percentage is represented on the y-axis. The x-axis denotes the response selected by the generation-Y medical students. A=students using social media application once a week for their medical education; B=students using social medical application two times a week for their medical education; C=students using social medical application three times a week for their medical education; D=students using social medical application daily for their medical education (note: a total of 75 undergraduate medical students participated in this study, of which eight were excluded, as six of them did not complete the questionnaire and other two declared that they were not using social media application in their medical education).

As shown in Figure 2, a majority (30/67, 45%) of generation-Y medical students used social media apps daily for their medical educational commitments. While designing the questionnaire, we assumed that by virtue of being generation-Y medical students, the participating respondents were regular users of different social media apps for noneducational activities (social interaction through group chat, blogging, information sharing, etc).

Therefore, our questionnaire focused on the use of social media apps by generation-Y medical students for educational purposes. The fact that there is a difference in the frequency of social media app use among the respondents in medical education most likely indicates a difference in their learning styles, which is common in medical schools and has been observed in other studies [38,39]. However, further elaborate studies need to be pursued to definitively confirm this conclusion.

The other aspect that may contribute to the observed difference is access to social media apps off-campus, because of various limiting factors such as restricted internet access and social pressure. However, as majority of our respondents spend 12-14 hours a day on campus (MBRU), where there is access to high-speed internet, we believe that the observed difference in the weekly use of social media app posits the likely differences in the learning styles of the respondents.

### Social Media App of Choice of Generation-Y Medical Students for Medical Education

In the questionnaire (Multimedia Appendix 1), we asked generation-Y medical students about the use of 16 different social media apps in their medical education. Social media app platforms to be included in the questionnaire were chosen based on ease of access (determined from gap analysis using available literature; Table 1); platforms that are commonly used in the region; and compatibility with both desktop and electronic smart devices such as tablets, phablets, and smartphones.

We did not include modules related to the virtual learning environment or learning management system such as Moodle [40] (github.com/moodle/moodle), Blackboard (Blackboard Inc, Washington DC), or Sakai (Brock University, St. Catharines, ON) in our list because these modules are not as user-friendly as mobile instant messaging service apps such as WhatsApp in terms of hosting and moderating extended discussion sessions [41,42]. In fact, at MBRU, students and instructors regularly use D2L (D2L Inc, Kitchener, ON), where study materials such as slides and clinical cases to be discussed are uploaded by the instructor for the students to access and download. However, when it comes to sharing videos or pursuing a discussion with regard to a specific topic, especially in courses where social media apps are used as a teaching tool, students prefer the use of an mobile instant messaging service. This was confirmed by the authors informally. Moreover, before the study, one of the authors, who is a year 2 generation-Y medical student, informally enquired with the year 1 and year 2 students if they had access to at least one of the abovementioned electronic smart devices. All year 1 and year 2 students had access to one or more of the electronic smart devices.

**Figure 3:**
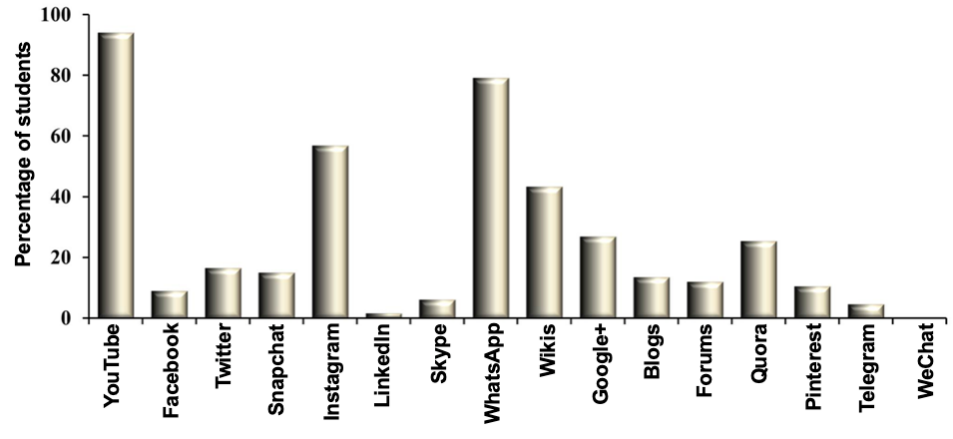
Comparison between different types of social media applications used by the students. Number of students in percentage is represented on the y-axis. x-axis denotes the response selected by the generation-Y medical students. (Note. YouTube was the most preferred social media application followed by WhatsApp among students).

Among the 16 social media apps platforms, YouTube and WhatsApp were the generation-Y medical students’ social media apps of preference for their medical education, as more than 50% of the respondents recurrently selected these social media app platforms (Figure 3).

**Figure 4:**
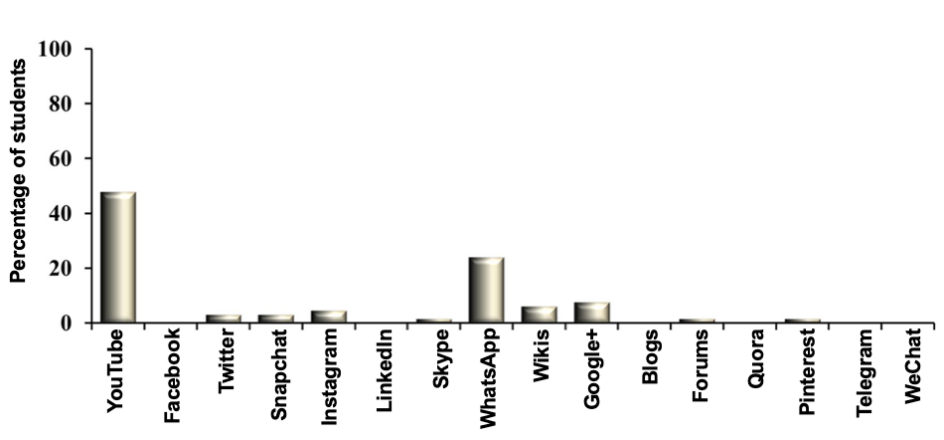
Frequency of use of a specific social media application platform in medical education by the generation-Y medical students.

We also investigated the frequency with which the respondents used a specific social media app. YouTube was the most preferred social media app, used most frequently by 48% of generation-Y medical students (Figure 4), followed by WhatsApp, which 23% of generation-Y medical students used in their medical education (Figure 4).

It is understandable that the use of YouTube is more frequent than WhatsApp. YouTube is the largest video-sharing platform on the internet, exceeding 1 billion users, with more than 300 hours of videos uploaded every minute [43]. Students in their preclinical years use it as a vital resource, in particular, for laying down foundations of basic sciences as well as obtaining insight into practical skills [44]. WhatsApp, on the other hand, is a platform for interactive discussion on a specific topic once the students are aware of the basic facts and figures pertaining to the topic [45].

The fact that generation-Y medical students exhibited a strong inclination to use YouTube provides a strong advantage to instructors who intend to integrate social media apps in teaching. It is well known that instructors delivering a traditional lecture with loads of content-heavy PowerPoint slides may confound what they teach with what students learn: The fact that an instructor has presented a specific segment of information does not necessarily mean that students have learned and assimilated that specific segment of information. In fact, cognitive load theory suggests that a learner’s brain is restricted in the volume of information it can process at a time [46]. Due to these limitations, innovative pedagogical strategies have been introduced, one of which is the *Flipped Lecture* or the *Flipped Classroom* technique. In the Flipped Classroom technique, students prepare for class by performing prework outside the class, frequently in the form of a video lecture or screencast. They then attend a session to work out practice problems and important clinical cases, engage in group work, and gain know-how with researching answers in a mentored environment [47].

For a student, the *Flipped Classroom* technique provides a strategy, where learning is self-paced (one can go over a recorded lecture as many times as required) and promotes the development of metacognitive skills through group work and peer-assisted learning [48]. For the instructor, however, the *Flipped Classroom* technique requires extensive preparation, as the lecture material needs to be organized and the instructor then needs to record the lecture for which suitable facilities and equipment are required (which is not often the case, especially in medical schools with restricted funding). Furthermore, as medicine is an evolving science, one-time recording of lectures is not a practical solution. To resolve these limitations, YouTube provides a practical, cost-effective solution. One of the feasible strategies (elaborated below) is to direct students to specific YouTube videos related to specific content and then have small group discussions promoted through the use of clinical vignettes and scenarios. Such activities can be structured further through the use of instructional design strategies of Gagne and Peyton [49-53], as we have designed and successfully implemented recently in the preclinical phase at MBRU [54]. In brief, incorporating YouTube in designing pedagogical strategies can provide students with a learning experience similar to the *Flipped Classroom* technique, concomitantly easing implementation of social media apps in a course for the instructor. In fact, in a study comparing the content of standard textbooks, eMedicine (Medscape) articles, and YouTube videos on cardiovascular mechanism, Azer et al showed that YouTube videos surpassed not only on the user interface front but also the content and integration of information athwart molecular and clinical levels [55]. However, it is pivotal that the instructor vets a specific YouTube video before recommending it to students, as studies show that there are numerous videos on YouTube that are erroneous with no regulation of content [56].

### Gender and Social Media App Preference

YouTube was the most preferred social media app among both male and female generation-Y medical students (Table 2).

**Table 2:** Preference of the 16 social media apps among generation-Y medical students.

Social media app	Female students, n (%)	Male students, n (%)
YouTube	23 (48)	9 (47)
Facebook	0 (0)	0 (0)
Twitter	2 (4)	0 (0)
Snapchat	2 (4)	0 (0)
Instagram	1 (2)	2 (10)
LinkedIn	0 (0)	0 (0)
Skype	0 (0)	9 (5)
WhatsApp	10 (20)	19 (31)
Wikis	4 (8)	0 (0)
Google+	5 (10)	0 (0)
Blogs	0 (0)	0 (0)
Forums	0 (0)	1 (5)
Quora	0 (0)	0 (0)
Pinterest	1 (2)	0 (0)
Telegram	0 (0)	0 (0)
WeChat	0 (0)	0 (0)

However, more diversity in the use of social media apps was observed in the female population, where the use of 8 of the 16 social media apps (YouTube, Twitter, Snapchat, Instagram, WhatsApp, Wiki, Google^+^, and Pinterest) was observed, whereas the male population used only 5 of the 16 social media app platforms (YouTube, Instagram, Skype, WhatsApp, and Forums).

Studies have shown that gender differences exist and affect how individuals engage in day-to-day activities. In fact, significant gender differences are observed between how men and women adopt and use technology [57,58]. A study by Chun has shown that women generally make comments and contributions that are more descriptive and lengthier when expressing themselves for knowledge management over different social media app platforms. Furthermore, women more frequently attempt to associate and integrate existing knowledge with the knowledge that they obtained during online discussions, thus creating new knowledge. The study also showed that the reason for the elaborate and more detailed responses by women can be attributed to their social assertiveness, establishing relationships and networks with other users of social media apps for knowledge management, thereby establishing a comfortable knowledge-sharing milieu with users with whom they had an established relationship [59]. Therefore, the use of social media app modules such as Snapchat, Instagram, and Pinterest is more common in women, as these modules support networking through elaborate discussions.

This finding has significant ramifications with regard to medical education in the region. Many medical schools, especially those in Saudi Arabia, have separate pedagogical sessions for male and female students [60]. On the basis of our findings, it can be concluded that a more diverse social media app–integrated pedagogical approach may appeal more to women than to men.

### Generation-Y Medical Students’ Perception of the Use of Social Media Apps in Mohammed Bin Rashid University

**Figure 5:**
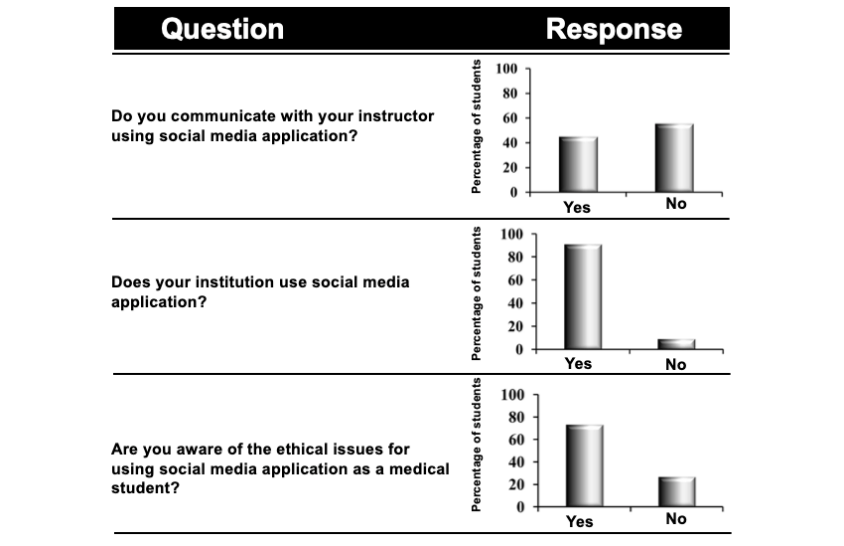
Generation-Y medical students’ perception about the use of social media application in the institution.

Generation-Y medical students’ perception of the use of social media apps at MBRU was investigated using three items (Figure 5). These items were polar questions requiring the respondent to indicate Yes or No, in line with one’s perception.

#### Effect of Social Media Apps on Communication Between Generation-Y Medical Students and Instructors

Only 37% of generation-Y medical students communicated with their instructors using social media apps (Figure 5). Most of the students used social media apps for their studies (Figure 3) on a frequent basis (Figure 2); the observed decrease in use may be because of the “digital divide” existing between generation-Y medical students and instructors, which has been observed in other studies as well [61,62]. Interestingly, more female than male students used social media apps to communicate with instructors (Table 3), which is in line with the social assertiveness of women elaborated above.

#### Students’ Perception of Institutional Use of Social Media Apps and Their Ethical Awareness

**Figure 6:**
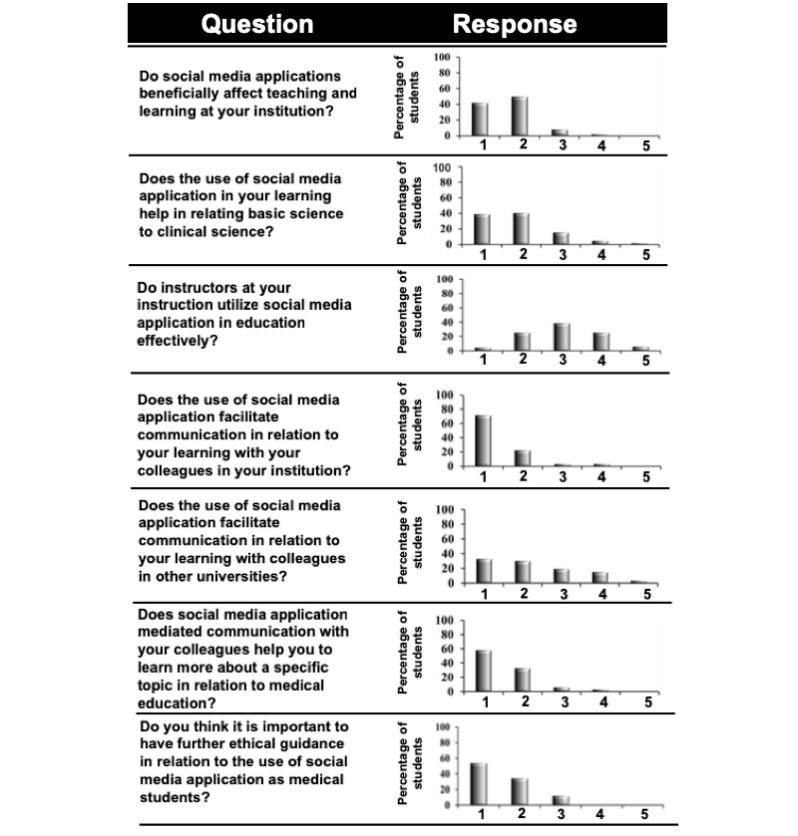
Response of generation-Y medical students to the Likert-scale questions used in the study.1=Strongly agree; 2=Agree; 3=Neutral; 4=Disagree; 5=Strongly disagree.

Most generation-Y medical students (91%) indicated that the institution was employing social media apps for supporting their education (Figure 5). As patient-centered health care, social media, and the internet are closely associated in health care in the 21st century [63], we decided to investigate if generation-Y medical students are aware of ethical issues such as confidentiality and privacy, associated with the use of social media apps. Most generation-Y medical students (49/67, 73%) provided a positive response. However, as ethics is a critical domain in medicine and health care, we believe that instructors should stress on this aspect before engaging students in social media app–driven pedagogy. This conclusion is also stressed upon because in the questionnaire, we included an item, “Do you think it is important to have ethical guidance for using social media as medical students?” for which the majority of generation-Y medical students requested further guidance (Figure 6).

Furthermore, when we grouped ethical guidance awareness of generation-Y medical students according to gender (Table 4), we found that more female than male students were aware of the ethical considerations associated with social media app use. This may be because of the increased social assertiveness exhibited by female generation-Y medical students, as observed and elaborated above.

### Generation-Y Medical Students’ Perception of the Effect of Social Media Apps on Medical Education

To assess generation-Y medical students’ perception on the effect of social media apps on medical education, we included 7 items in the questionnaire in the form of Likert scale questions (Figure 6). As indicated earlier, as ethical considerations associated with the use of social media apps are important, one of these 7 items reconfirmed if the generation-Y medical students required further guidance on the use of social media apps in medical education. Majority of the generation-Y medical students responded positively (Figure 6). This observation supports our abovementioned affirmation regarding instructors providing a preamble to generation-Y medical students about the ethical contemplations associated with the use of social media apps in medical education and health care.

#### Effect of Social Media Apps on Teaching and Learning

Item 8 in the questionnaire explored generation-Y medical students’ perception about whether social media apps influenced teaching and learning positively. Majority (91%) of the generation-Y medical students concurred that social media apps constructively affected their scholarship. On categorization of responses according to gender, no significant difference was observed between female and male students (Table 5). This alludes to our early certitude of “digital divide,” although both female and male generation-Y medical students used social media apps regularly (Figures 2 and 3) and considered social media apps to benefit their erudition (Figure 6). As compared to male generation-Y medical students, female students interacted with instructors more often using social media apps, because of the latter’s higher and diverse electronic social presence (Table 2).

**Table 3:** Gender preference in the use of social media apps for communication between generation-Y medical students and instructors.

Response	Female students, n (%)	Male students, n (%)
Yes	23 (48)	7 (37)
No	25 (52)	12 (63)

**Table 4:** Ethical guidance awareness associated with the use of social media apps, categorized according to gender.

Response	Female students, n (%)	Male students, n (%)
Yes	38 (79)	11 (57)
No	10 (21)	8 (43)

Interestingly, 5.3% of male generation-Y medical students perceived social media apps to have a detrimental effect on teaching and learning. “Digitally Shy” learners have been found to learn better when they are able to visually assert the expressions (facial expressions, body language, etc) of the instructor/fellow student during the learning process; in other words, these students learn better when they are emotionally involved [64]. Although a small proportion of generation-Y medical students in our study belong to this category, specific measures should be implemented to cater to the learning needs of such students. One of the ways is to blend social media app–integrated pedagogy with small-group teaching using team-based or problem-based approaches.

#### Generation-Y Medical Students’ Perceptions of the Effect of Social Media Apps on Bridging the Basic Science-Clinical Science Divide

One of the key aspects of Flexnerism is that conventional basic sciences are considered the foundational sciences upon which the groundwork for medical practice is centered. The beneficence of the basic sciences to the so-called “ontogenesis of the medical practitioner” goes beyond mere accretion of factual information and serves to inform the critical thinking and decisional framework. In fact, clinical medicine is based upon the identification, categorization, and subsequent management/treatment of abnormal physiology (pathophysiology). However, most often, the pedagogical techniques employed in meeting the learning objectives of the basic science courses fail to identify strategies that can integrate these “facts and figures” to rationally solve clinical problems presented by patients, although they motivate students to rote memorize a corpus of clinically relevant “facts and figures.” Does the use of social media apps by generation-Y medical students in medical education bridge the so-called “Basic Science-Clinical Science divide”? As indicated in Figure 6 and Table 6, a majority of respondents concurred that the use of social media apps helped in relating basic science to clinical science. Approximately 20% (13/67) of both female and male students found that the use of social media apps did not facilitate bridging the basic science-clinical science divide. This may be attributed to the type of social media app platforms that these students used for their medical education as well as the different learning styles of students in a medical school [65].

#### Generation-Y Medical Students’ Perception of Instructors Integrating Social Media App in Teaching

One of the items in the questionnaire enquired about generation-Y medical students’ perception of integration of social media apps by instructors at MBRU while teaching. A mixed response was observed (Figure 6). To obtain a better insight, we grouped the responses according to gender, where also a similar trend was noted (Table 7). MBRU is a nascent institution, and many of the faculty members, especially in the basic sciences, do not have extensive teaching experience, which may account for the low use of social media apps platforms in teaching. In addition, junior faculty members are often under pressure of clinical and research productivity, and as such, are un able to invest adequate time for the development of innovative pedagogical strategies. Our observations are in line with the results of Kim et al [66]. The other reason that may account for the mixed response may be attributed to faculty members’ lack of understanding of how generation-Y medical students employ social media apps platforms in medical education, which may hinder integration of the generation-Y medical students–preferred social media app platforms in their pedagogical strategies/techniques, in turn creating a so-called “digital divide” between the generation-Y medical students and faculty members. Often, pedagogical approaches of instructors affect students’ learning approaches [67]. Trigwell et al found that in classes where instructors portrayed their method to pedagogy as transferring knowledge, students were more likely to report surface learning approaches [68]. However, in a student-centered curriculum (as the one at MBRU), students have more responsibility toward their own erudition (what and how) [69]; therefore, pedagogical approaches that appeal to generation-Y medical students need to be designed. Both the above aspects indicate the need for a streamlined faculty-development program. One effective way will be to institute a faculty mentorship program per one of the models proposed by Lancaster et al [70].

**Table 5:** Generation-Y medical students’ perception of the effect of social media apps on teaching and learning.

Response	Female students, n (%)	Male students, n (%)
Positive (strongly agree and agree)	44 (92)	17 (90)
Neutral	4 (8)	1 (5)
Negative (strongly disagree and disagree)	0 (0)	1 (5)

**Table 6:** Generation-Y medical students’ perceptions of the role of social media apps in connecting basic science to clinical science.

Response	Female students, n (%)	Male students, n (%)
Positive (strongly agree and agree)	38 (79)	15 (79)
Neutral	7 (15)	3 (16)
Negative (strongly disagree and disagree)	3 (6)	1 (5)

**Table 7:** Generation-Y medical students’ perception of instructors integrating social media apps in teaching.

Response	Female students, n (%)	Male students, n (%)
Positive (strongly agree and agree)	13 (27)	7 (37)
Neutral	21 (44)	5 (26)
Negative (strongly disagree and disagree)	14 (29)	7 (37)

#### Generation-Y Medical Students’ Perceptions of the Use of Social Media Apps for Augmenting Peer-Assisted Learning

Peer-assisted learning propositions a valuable technique to augment the learning experience in medical school [71] and has been shown to remedy areas of weakness in knowledge and competencies, deliver a safe milieu for practice and strengthening of curriculum content, and nurture a sense of community among junior and senior peers [72]. Furthermore, peer-assisted learning is an efficient approach to engage students beyond a superficial level [73]. This “deep” approach to pedagogy is largely due to the aim of peer-assisted learning, which should be supplementary to, rather than separate from, existing pedagogical strategies [74]. The method is further aided by the ability of this approach to be more interactive [75], more targeted toward identified areas of interest or weakness [76], and less authoritative than traditional pedagogical strategies. What strengthens these characteristics of the teaching process is the superior understanding that senior peers have of the learning requirements and competencies of junior students as well as the curriculum and assessment necessities, compared with highly trained and veteran senior consultants. This propinquity in understanding defines the valuable social and cognitive analogy that senior students offer. This may contribute not only to students’ appreciation of being trained by peers [77] but also to the critical mass and depth of learning achievable by a student when provided with suitable instruction, as indicated by Vygotsky’s Zone of Proximal Development [78].

In line with the abovementioned benefits of peer-assisted learning, we wanted to assess generation-Y medical students’ perspectives of whether social media apps augment peer-assisted learning in their medical education. Almost all students (both male and female; Table 8) strongly agreed that social media app platforms assisted them in their learning process by instituting an effective channel of communication between them and their colleagues. However, few female students disagreed on this aspect (Table 8). This may be attributed to social loafing, which is often observed in collaborative group activities/discussions [79]. However, further research is essential to determine whether instances of social loafing are observed in peer-assisted learning in undergraduate medical education as well as the effects of social loafing during peer-assisted learning on students’ performance and whether this occurs across a range of group sizes.

#### Generation-Y Medical Students’ Perception of the Use of Social Media Apps for Networking

Networking is an essential aspect of medical practice. In fact, doctors typically refer their patients to specialists that they know, creating an unofficial network. These so-called “informal networks” are pivotal to the excellence and cost of care that a patient receives from any individual doctor, hospital, or medical group. In a study employing 987,000 Medicare beneficiaries aged 65 years or older [80], across five states—Ohio, Pennsylvania, Tennessee, Washington, and Wisconsin—in the United States, Lawrence et al aimed to identify physician networks; to determine whether the rate of ambulatory care-sensitive hospital admissions varies across networks, even different networks at the same hospital; and to examine the relationship between ambulatory care-sensitive hospital admission rates and network characteristics.

The study identified 417 such informal networks, with a mean size of 129 physicians, and found that the rate of ambulatory care-sensitive hospital admissions (ie, potentially avoidable admissions of patients with chronic diseases such as congestive heart failure and asthma) varied significantly across the networks. Thus, it is essential to highlight the importance of effective networking to medical students.

As social media app platforms augment networking between generation-Y medical students at MBRU in relation to their medical education, we aimed to investigate if such networking was founded/strengthened between generation-Y medical students at MBRU and those at other universities as well as to appraise whether social media app platforms can be an effective mode for establishing informal networks among generation-Y medical students across different curricula. A mixed response was observed: The response varied widely between male and female students (Table 9).

**Table 8:** Generation-Y medical students’ perceptions of the use of social media app for intrauniversity communication.

Response	Female students, n (%)	Male students, n (%)
Positive (strongly agree and agree)	45 (94)	18 (95)
Neutral	1 (2)	1 (5)
Negative (strongly disagree and disagree)	2 (4)	0 (0)

**Table 9:** Generation-Y medical students’ perceptions of the use of social media apps for interuniversity communication.

Response	Female students, n (%)	Male students, n (%)
Positive (strongly agree and agree)	40 (58)	14 (74)
Neutral	14 (21)	3 (16)
Negative (strongly disagree and disagree)	14 (21)	2 (10)

**Figure 7:**
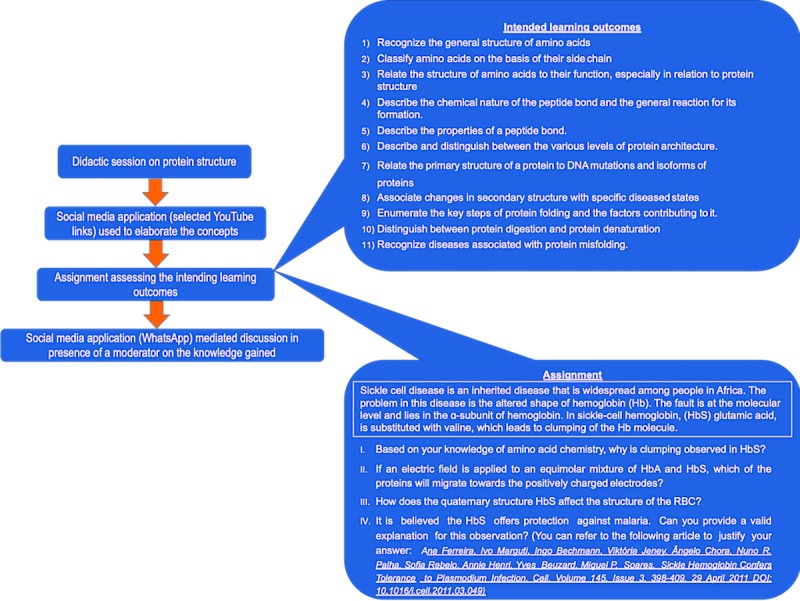
An example of social media application mediated delivery of a topic in biochemistry.(Note, the strategy for the delivery was designed using the social medial applications preferred by generation-Y medical students).

The number of female and male students effectively using social media apps to communicate with their colleagues in other universities was 58% and 73%, respectively. The primary reason for such a diverse response may be attributed to the fact that as MBRU is a new institution in the region, it still does not have an established digital footprint to initiate a focal point for establishing informal networks.

Moreover, the reason for a greater proportion of males using social media apps effectively for interuniversity networking (Table 9) may be that at MBRU, most of the male students are from countries outside the United Arab Emirates; hence, these students may be networking with their peers in medical schools from their countries of origin, to discuss various facets of medical education. Thus, dedicated research is required to provide more detailed insight into this aspect.

### Simple Strategy to Employ Social Media Apps in Pedagogy

In this study, we also delineated a simple strategy to implement social media apps in a pedagogy of generation-Y medical students. We employed a social media app–integrated pedagogical strategy to deliver the intended learning outcomes with regard to the topic *Amino Acids and Protein Structure* in the course of Enzymes and Metabolism, which is a 3-credit Biochemistry course in the undergraduate medical curriculum at MBRU.

The entire cohort of 45 students registered for the course, which spanned over 15 weeks, in which the *Amino Acids and Protein Structure* topic was delivered over a period of 3 weeks. Figure 7 demonstrates the steps used to integrate social media apps in disseminating information on the topic. A didactic session of 20 min was used to deliver the intended learning outcomes pertaining to the *Amino Acids and Protein Structure* topic (Figure 7).

The students were then guided to online resources (YouTube videos) selected by the instructor to further expatiate on the topic and were provided with an assignment on sickle disease (Figure 7), for which relevant items were used to assess the intended learning outcomes.

The assignment was part of the formative assessment of the course and was graded using a defined grading rubric. As the students prepared the assignment, queries and doubts were addressed in a discussion group established on a social media app platform (WhatsApp). Students had the liberty of adding a participant to the group; however, the discussion threads were monitored by the instructor. Following the completion of the assignment, general feedback was provided to the entire cohort through the discussion group.

Although the strategy still needs to be formally evaluated, the Enzymes and Metabolism course received a positive feedback from the cohort, with approximately 93% of the students strongly supporting the teaching style. Furthermore, in informal interactions with the instructors, the students indicated that integration of social media apps in teaching Biochemistry facilitated a better understanding of the delivered concepts.

## Discussion

### Limitations and Future Directions

Our study has a few specific limitations that are discussed below:

We used a prevalidated questionnaire [25] with minor modifications. However, MBRU caters to students from 19 different nationalities from different high school curricula. Therefore, it would have been ideal to develop a questionnaire following a needs-assessment study. An educational needs assessment can be defined as the gap between “what is known” and “what should be known” [81]. We performed a general needs assessment using *gap analysis* [82] through a literature review of seminal articles. Our search identified five recent systematic reviews investigating the use and role of social media apps in undergraduate medical education (Table 1). Observations from these systematic reviews were used for modifying the prevalidated questionnaire (Multimedia Appendix 1). However, since our study was a proof-of-concept study, it lacked a needs assessment of targeted learners.

One of our future goals is to employ one of the consensus methods—nominal group technique or Delphi Technique—to develop a framework for needs assessment of targeted learners. Both these methods intend to attain a concourse of views surrounding a particular topic. *The definitive nominal group technique* has 4 phases—silent generation, round robin, clarification, and voting (ranking) [83]. The *Delphi technique,* on the other hand, employs a multistage self-completed survey with individual feedback, to define agreement from a bigger group of “experts” [84]. Through the concerted application of these techniques, one would be able to identify novel domains in generation-Y medical students’ education, where social media apps can be employed. Questions pertaining to these domains can be incorporated into the existing questionnaire to obtain more extensive responses. Development and validation of such a framework can form the basis of future studies.

Our study has identified social media apps that are preferred by generation-Y medical students. Furthermore, we employed these social media apps modules in disseminating specific intended learning outcomes, which received positive feedback from the learners. However, successful incorporation of these social media apps in the design of pedagogical strategies concomitantly with curriculum and course design still needs to be appraised, for which an elaborate study needs to be strategized. One of the ways to strategize this study is to design a lesson plan using social media apps employing conventional instructional design strategies, such as that of Gagne and Peyton [85], followed by incorporation of these lesson plans into courses founded on Kern’s 6-step approach [86]; these courses can then be evaluated by established models of evaluation such as the Stake’s Congruence-Contingency Model [87]. Thus, the quality of these social media app–incorporated courses can be perfected using innovative quality assurance and quality-enhancement frameworks strategized according to the Deming cycle, customized to the ISO 9001:2000 standards in medical education [88].

Our study targets generation-Y medical students who have transitioned from high school to the university, which happens mostly in middle schools located in the Middle East and North Africa. Although the observations in our study can be translated into the design of effective social media app–integrated pedagogical strategies for medical schools in the Middle East and North Africa region, they may be insufficient for translation to medical schools where generation-Y medical students often attend junior college before transiting to the university, because the learning styles and cognitive and metacognitive abilities of both these group of students are significantly different [89].

Our study focuses on generation-Y medical students who are in the preclinical phase in the medical curriculum, where the emphasis is on dissemination of knowledge and provision of a basic know-how of this knowledge through this app, addressing basic clinical problems in the form of simple vignettes/cases. Therefore, if social media apps are to be employed in the design of pedagogical strategies implemented in the clinical phase of the undergraduate medical curriculum, a separate study needs to be strategized, as the goal of the clinical phase is to apply the knowledge acquired in the preclinical phase to diagnose and manage complex clinical problems as well as to grow into a safe and competent health practitioner.

Although, in designing the questionnaire, we attempted to include all the popular social media app modules, we omitted a few such as Tumblr and Reddit. This is because these social media app modules are either restricted for use in the region or are not popular among end users in the region.

### Conclusions

This proof-of-concept study identifies social media app platforms preferred by generation-Y medical students in medical education, specifically in a spiral curriculum. In addition, this study identifies the differences between female and male generation-Y medical students in terms of the use of social media apps in medical education, which we believe will assist instructors in designing pedagogical strategies to integrate social media apps. Furthermore, we have determined the perception of generation-Y medical students on the implementation of social media apps in medical education, which we believe will facilitate the design of more student-centered pedagogical approaches. A simple strategy to implement social media apps in teaching generation-Y medical students is also presented, which can be translated to any course in the preclinical phase of the medical curriculum. Furthermore, the identified limitations of this study provide an understanding of the gaps in research on integration of social media apps in a medical curriculum catering to generation-Y medical students. In other words, 21st century medical education requires effective use of social media app platforms to augment competency-based medical education: *Augmentation of Flexnerism in the current scenario is possible only by the adaptation of Twitterism*.

## Appendix

Multimedia Appendix 1 Questionnaire.

Multimedia Appendix 2 Reviewer comments.

## Abbreviations

MBRU: Mohammed Bin Rashid University
