# Supplementary material for: Augmenting Flexnerism Via Twitterism: Need for Integrating Social Media Application in Blueprinting Pedagogical Strategies for Undergraduate Medical Education
Source: JMIR Med Educ. 2019 Mar 25;5(1):e12403. doi: 10.2196/12403 (PMC6452274; doi:10.2196/12403)
Supplement: Multimedia Appendix 1 [file mededu_v5i1e12403_app1.pdf]

Supplementary Information

Augmenting Flexnerism via Twitterism: Need for Integrating Social Media Application in Blueprinting Pedagogical Strategies for Undergraduate Medical Education

Yajnavalka Banerjee [1, 5], *, Ph.D., Tambi Richa [1,2, #] Ph. D.; Mandana Gholami [3, #]; Alawi A. Alsheikh-Ali [1] MD; Peter J Lansberg [4] MD, Ph.D.;

[1]Department of Basic Medical Sciences, Mohammed Bin Rashid University of Medicine and Health Sciences (MBRU), Academic Medical Center (AMC), Dubai Health Care City (DHCC), Dubai- 505055, United Arab Emirates (AE)

[2]Laboratory for Computational Molecular Design, RIKEN Center for Biosystems Dynamics Research (BDR), Osaka 5650874, Japan

[3]Bachelor of Medicine & Bachelor of Surgery (MBBS) program, Mohammed Bin Rashid University of Medicine and Health Sciences (MBRU), Academic Medical Center, Dubai Health Care City (DHCC), Dubai-505055, United Arab Emirates (AE)

[4]Department of Pediatrics, Section Molecular Genetics, University Medical Center Groningen, Building 3226, Room 04.14, Internal Zip Code EA12, Antonius Deusinglaan 19713 AV Groningen, The Netherlands

[5]Masters in Medical Education (MMed) Program, Center Medical Education, University of Dundee, Nethergate, Dundee, DD14HN, Scotland, United Kingdom

[#] These authors have contributed equally to the research depicted in this manuscript.

[*]**Address all correspondences to**:

Dr. Yajnavalka Banerjee, MBRU, AMC, DHCC, Dubai – 505055, UAE

E-mail: yaj.banerjee[at]gmail.com OR Yajnavalka.Banerjee[at]mbru.ac.ae OR YBanerjee[at]Dundee.ac.uk

Telephone: +971-0568345125

Appendix 1

Dear respondent,

Research is being pursued to explore the ‘*Role of Social Media on Undergraduate Medical Education*’.

This study aims to identify social media application (SMA) module(s) which can be integrated in designing and strategizing innovative teaching strategies. In order to do so, a questionnaire is being circulated, directed to MBBS undergraduate students at MBRU. All the items (questions) relate to your experience in medical education at MBRU. Your co-operation in taking the time to participate in this research is highly appreciated.

Kindly note that it may take several minutes to fill out the questionnaire.

Please note that the answers to this questionnaire will be utilized for the purpose of this research only, and no participant information will be disclosed.

Your time and effort are much appreciated in advance.

Sincere Regards.

Respondent ID ……………..

Following questions were displayed only if the respondent consented to participating in the study

1. Do you consent to proceed with the following questionnaire?

☐Yes ☐No

2. Gender:

☐Male ☐Female

3. Medical Year

☐Year 1 ☐Year 2

4. Do you use social media applications (SMA) in your education?

☐Yes ☐No

5. How frequently do you use social media in your learning?

☐Weekly ☐Twice a week ☐Three times a week ☐Daily

6. Which of the following social media applications are you using in your education? (Note: You can select multiple)

☐Youtube ☐Facebook ☐Twitter ☐Snapchat ☐Instagram

☐Linkedin ☐Skype ☐Whatsapp ☐Wikis ☐Blogs

☐Google+ ☐Blogs ☐Forums Others ______________

7. Which one of SMAs are you using the most for your education?

☐Youtube ☐Facebook ☐Twitter ☐Snapchat ☐Instagram

☐Linkedin ☐Skype ☐Whatsapp ☐Wikis ☐Blogs

☐Google+ ☐Blogs ☐Forums Others___________

8. Do SMA beneficially affect teaching and learning at your institution?

☐Strongly agree ☐Agree ☐Neutral ☐Disagree ☐Strongly Disagree

9. Does the use of SMA in your learning help in relating to basic science to clinical science?

☐Strongly agree ☐Agree ☐Neutral ☐Disagree ☐Strongly Disagree

10. Do you communicate with your instructor using SMA?

☐Yes ☐No

11. Do instructors at your institution utilize SMA in education effectively?

☐Strongly agree ☐Agree ☐Neutral ☐Disagree ☐Strongly Disagree

12. Does your institution use SMA?

☐Yes ☐No

13. Does the use of SMA facilitate communication in relation to your learning with your colleagues in your institution?

☐Strongly agree ☐Agree ☐Neutral ☐Disagree ☐Strongly Disagree

14. Does SMA facilitate communication in relation to your learning with colleagues in other universities?

☐Strongly agree ☐Agree ☐Neutral ☐Disagree ☐Strongly Disagree

15. Does SMA mediated communication with your colleagues help you to learn more about a specific topic in relation to medical education?

☐Strongly agree ☐Agree ☐Neutral ☐Disagree ☐Strongly Disagree

16. Are you aware of the ethical issues for using SMA as a medical student?

☐Yes ☐No

17. Do you think it is important to have further ethical guidance in relation to the use of SMA as medical students?

☐ Strongly agree ☐ Agree ☐ Neutral ☐ Disagree ☐ Strongly Disagree

17. Observations/Comments:

Appendix 2

Certain GYMS had specific comments with regards to the use of SMA in Medical Education. These were indicated in the comment box in the questionnaire:

1. SMA should be used in medical education for teaching

2. Ethical considerations associated and learning with the use of SMA should be kept in mind.

3. One of the GYMS advocated that Reddit platform should be used more in comparison to other platforms.

4. One of the GYMS highlighted that more such studies focusing on education should be pursued.

In summary GYMS acknowledged the importance of this study and stressed on integrating SMA in their education at the institution.
