# Supplementary material for: Augmenting Flexnerism Via Twitterism: Need for Integrating Social Media Application in Blueprinting Pedagogical Strategies for Undergraduate Medical Education
Source: JMIR Med Educ. 2019 Mar 25;5(1):e12403. doi: 10.2196/12403 (PMC6452274; doi:10.2196/12403)
Supplement: Multimedia Appendix 2 [file mededu_v5i1e12403_app2.pdf]

**Authors' Response to Peer Reviewer's Comments**

The changes introduced in the manuscript in line with reviewer's comments are indicated using track changes, which I believe will allow both the reviewer and the editorial team of JMIR Medical Education to follow the changes that have been introduced in the revised manuscript.

REVIEWER AO:
GENERAL COMMENTS
=============
THIS PAPER IS VERY INTERESTING AND WELL WRITTEN; HOWEVER, IT HAS A LOT OF EXTRANEOUS INFORMATION THAT COULD BE POTENTIALLY REMOVED. I LOVE THAT THIS PAPER COVERS SOCIAL MEDIA IN MEDICAL EDUCATION, THIS IS AN IMPORTANT BUT NOT FREQUENTLY COVERED TOPIC. I LIKE THE GENDER TWIST. OVERALL, I THINK THE PAPER IS A LITTLE LONG AND COULD POTENTIALLY BE BROKEN DOWN INTO 2-3 SMALLER PAPERS--HOWEVER IT IS FINE IN ITS ENTIRETY.

RESPONSE

First, we will like to thank the reviewer for the positive and stimulating comment.

The manuscript was drafted in a way such that the study observations are supported by learning theories, where ever applicable. Further, our attempt has also been to provide the reader with snippets and insights from similar studies in literature, which as the reviewer has identified "isn't a frequently covered topic". This added to the bulk of the manuscript.
Therefore, we have edited specific sections of the manuscript to reduce the number of words, but in a way such that the flow, understandability and readability of the manuscript isn't affected. The previous version of the manuscript contained 8080 words in text, whereas the revised version now has 7317 words (reduction of 763 words). These edits and changes introduced in the revised version of the manuscript are indicated using track changes.
We will like the manuscript to be published as a single article, without breaking it down into 2 or 3 separate articles, as in its present form the manuscript provides the reader with a comprehensive review highlighting the gaps with regards to the use of social media application (SMA) in the design of pedagogical strategies, and dissemination of knowledge in undergraduate medical education. This we believe will provide impetus for initiating new research in the domain of "SMA integrated pedagogical strategies" in medical education, concomitantly increasing the number of sites for the article.

SPECIFIC COMMENTS
=============
MAJOR COMMENTS
---------------------
1. WELL WRITTEN, I GREATLY ENJOYED THE GENDER TWIST

RESPONSE
We thank the reviewer for the encouraging and motivating insight.

2. INTERESTING TOPIC THAT NEEDS TO BE FURTHER RESEARCHED, THIS PAPER DOES A GREAT JOB DIVING DEEPER INTO THE TOPIC AND PROVIDING ONE UNIVERSITY'S INFORMATION ON THE TOPIC. THE PAPER IS A LITTLE DENSE AND COULD POTENTIALLY BE SHORTENED.

RESPONSE
We thank the reviewer for the comment. We do concur that the manuscript in comparison to a normal article is a bit lengthy. In the revised version we have edited specific sections of the manuscript in a way such that the flow, understandability and readability of the manuscript isn't affected. The previous version of the manuscript contained 8080 words in text, whereas the revised version now has 7317 words (reduction of 763 words). These edits and changes introduced in the revised version of the manuscript are indicated using track changes.
Further as indicated in our response to GENERAL COMMENTS, in its present form the manuscript provides the reader with a comprehensive review highlighting the gaps with regards to the use of social media application (SMA) in the design of pedagogical strategies, and dissemination of knowledge in undergraduate medical education. This we believe will provide impetus for initiating new research in the domain of "SMA integrated pedagogical strategies" in medical education. This endeavor although has also added to the bulk of the manuscript, but we firmly believe will be of benefit to the medical education community.

3. WELL THOUGHT-OUT LIMITATIONS.

RESPONSE
We thank the reviewer for the positive and inspiring insight.

MINOR COMMENTS
---------------------
4. NONE

We firmly believe that we have addressed the reviewer's comments to the best our abilities.

Please do not hesitate to communicate with me, if your office requires further information or clarification

The manuscript was fast-tracked for review.

Looking forward to receiving a positive response from your side at the earliest.

Thanking you for your time and Kind Consideration,

Regards and Best Wishes

Yajnavalka Banerjee

Corresponding Author
